# MRI background parenchymal enhancement, breast density and breast cancer risk factors: A cross-sectional study in pre- and post-menopausal women

**DOI:** 10.1038/s41523-022-00458-2

**Published:** 2022-08-25

**Authors:** Jennifer D. Brooks, Rebecca A. G. Christensen, Janice S. Sung, Malcolm C. Pike, Irene Orlow, Jonine L. Bernstein, Elizabeth A. Morris

**Affiliations:** 1 Dalla Lana School of Public Health, University of Toronto, Toronto, Ontario Canada; 2 Department of Radiology, Memorial Sloan Kettering Cancer Center, New York, NY USA; 3 Department of Epidemiology and Biostatistics, Memorial Sloan Kettering Cancer Center, New York, NY USA; 4 Department of Radiology, University of California Davis, Sacramento, CA USA

**Keywords:** Risk factors, Cancer epidemiology, Breast cancer, Cancer screening, Breast cancer

## Abstract

Breast tissue enhances on contrast MRI and is called background parenchymal enhancement (BPE). Having high BPE has been associated with an increased risk of breast cancer. We examined the relationship between BPE and the amount of fibroglandular tissue on MRI (MRI-FGT) and breast cancer risk factors. This was a cross-sectional study of 415 women without breast cancer undergoing contrast-enhanced breast MRI at Memorial Sloan Kettering Cancer Center. All women completed a questionnaire assessing exposures at the time of MRI. Prevalence ratios (PR) and 95% confidence intervals (CI) describing the relationship between breast cancer risk factors and BPE and MRI-FGT were generated using modified Poisson regression. In multivariable-adjusted models a positive association between body mass index (BMI) and BPE was observed, with a 5-unit increase in BMI associated with a 14% and 44% increase in prevalence of high BPE in pre- and post-menopausal women, respectively. Conversely, a strong inverse relationship between BMI and MRI-FGT was observed in both pre- (PR = 0.66, 95% CI 0.57, 0.76) and post-menopausal (PR = 0.66, 95% CI 0.56, 0.78) women. Use of preventive medication (e.g., tamoxifen) was associated with having low BPE, while no association was observed for MRI-FGT. BPE is an imaging marker available from standard contrast-enhanced MRI, that is influenced by endogenous and exogenous hormonal exposures in both pre- and post-menopausal women.

## Introduction

Mammographic percent density (MPD) is a measure of the proportion of the normal breast occupied by fibroglandular tissue (FGT), seen as dense (white) areas on a mammogram. While MPD is considered to be one of the strongest, established risk factors for breast cancer^1^, there are other features (e.g., texture features) on mammogram that have also been implicated in risk^2^.

In the United States women at a high risk of breast cancer (i.e., ≥20% lifetime risk) are recommended to undergo annual screening with contrast-enhanced magnetic resonance imaging (MRI) in addition to mammography^3^. The amount of fibroglandular tissue in the breast can be assessed volumetrically on MRI (MRI-FGT) and is known to be correlated with MPD^4,5^. Much like MPD, MRI-FGT has been shown to be associated with breast cancer risk^6^, and sensitive to endogenous (e.g., menopause) and exogenous (e.g., tamoxifen, aromatase inhibitors) hormonal exposures^7–9^. Like mammogram, there are other image features from MRI that may be associated with breast cancer risk.

Contrast-enhanced MRI uses an intravenously injected contrast agent, to help visualize tumors through the identification of distinct patterns of contrast dispersion^10,11^. The MRI signal from normal FGT also enhances to varying degrees and is called background parenchymal enhancement (BPE). BPE is recorded as the proportion of FGT in the breast that enhances. Having high BPE has been associated with an increased risk of breast cancer in some^6,12–17^, but not all^18,19^ studies. Notably, this association is thought to be independent of MRI-FGT^6,16^. BPE has been shown to be highly influenced by both endogenous (e.g., menopausal status^7^, serum estrogen concentrations^20^, body mass index [BMI]^21,22^) and exogenous (e.g., menopausal hormone therapy [MHT]^23,24^, tamoxifen^9,25^, aromatase inhibitors^8,26^) hormonal exposures. However, many of these studies have been small or did not consider relevant confounders.

The objective of this study was to contribute to our understanding of BPE and MRI-FGT as imaging markers of breast cancer risk by examining their relationship with established breast cancer risk factors.

## Results

### Distribution of Breast Cancer Risk Factors

The median age at MRI was 49 years, 48% of women were postmenopausal, and 90% self-identified as White (Table 1). Most women (82% of premenopausal and 88% of postmenopausal), were having an MRI for high-risk breast cancer screening purposes. As expected, BPE and MRI-FGT were both higher in premenopausal than in postmenopausal women (*P* < 0.0001 for both) (Table 2). Weighted Cohen’s kappa coefficients for repeat reads of BPE and MRI-FGT were 0.87 (95% CI 0.76, 0.98) and 0.92 (95% CI 0.83, 1.00) (i.e., ‘almost perfect agreement’), respectively. Notably BPE and MRI-FGT were not correlated (Pearson correlation coefficient and p-value: postmenopausal women *r* = −0.00658, *p* = 0.93; premenopausal women: *r* = −0.09307 *p* = 0.17). The distribution of different breast cancer risk factors in the study population are shown in Tables 1 and 3.

**Table 1:** Characteristics of the study population by menopausal status at the time of MRI.

Patient characteristics, *N* (%)	Premenopausal (*N* = 217)	Postmenopausal (*N* = 198)
Age at MRI, median (range)	43 (25–58)	57 (39–77)
Reason for MRI^a^		
Abnormal screening mammogram	33 (15.0)	36 (18.1)
Lump in breast	23 (10.5)	13 (6.5)
High-risk breast cancer screening	180 (81.8)	175 (87.9)
Other	9 (4.1)	11 (5.5)
Race		
White/Caucasian	194 (88.2)	179 (90.0)
Black/African American	11 (5.0)	11 (5.5)
Asian or Pacific Islander	8 (3.6)	4 (2.0)
Other	7 (3.2)	5 (2.5)
Body mass index (BMI, kg/m^2^), median (range)	22.1 (17.7, 43.6)	23.8 (17.9, 50.1)
<18.5	9 (4.1)	5 (2.5)
18.5–<25	154 (70.0)	116 (58.3)
25–<30	38 (17.3)	43 (21.6)
≥30	19 (8.6)	35 (17.6)
Age at menarche		
Median^b^ (range)	12 (9–18)	13 (7–17)
<13 years	102 (46.4)	103 (51.8)
≥13 years	118 (53.6)	96 (48.2)
Parity		
Nulliparous	81 (36.8)	52 (26.1)
Parous	139 (63.2)	147 (73.9)
Number of full-term pregnancies		
Nulliparous	81 (36.8)	52 (26.1)
1	26 (11.8)	27 (13.6)
2	79 (35.9)	80 (40.2)
≥3	34 (15.5)	40 (20.1)
Age first full-term pregnancy (years)		
<25	10 (7.2)	25 (17.0)
25–<29	38 (27.3)	57 (38.8)
≥30	91 (65.5)	65 (44.2)

*MRI* magnetic resonance imagin, *BMI* body mass index, *BPE* background parenchymal enhancement, *MRI-FGT* amount of fibroglandular tissue on MRI, *ADH* atypical ductal hyperplasia, *ALH* atypical lobular hyperplasia, *LCIS* lobular carcinoma in situ, *NA* not applicable, *VUS* variants of unknown significance.

^a^Women were asked to indicate all that apply.

^b^Median age at menarche in the full study population was 13 years.

**Table 2:** Distribution of background parenchymal enhancement (BPE) and fibroglandular tissue (MRI-FGT) in pre- and postmenopausal women.

MRI Measurement, *N* (%)	Premenopausal (*N* = 217)	Postmenopausal (*N* = 198)
Background Parenchymal Enhancement (BPE)		
Minimal	42 (19.1)	109 (54.8)
Mild	102 (46.4)	66 (33.2)
Moderate	55 (25.0)	16 (8.0)
Marked	21 (9.5)	8 (4.0)
Fibroglandular Tissue (MRI-FGT)		
Predominantly fatty	7 (3.2)	24 (12.1)
Scattered densities	38 (17.3)	55 (27.6)
Heterogeneously dense	98 (44.5)	107 (53.8)
Extremely dense	77 (35.0)	13 (6.5)

**Table 3:** Distribution of breast cancer risk factors by menopausal status at the time of MRI.

Patient Characteristics, *N* (%)	Premenopausal (*N* = 217)	Postmenopausal (*N* = 198)
First degree family history of breast cancer		
No	56 (25.5)	60 (30.3)
Yes	164 (74.5)	138 (69.7)
*BRCA* mutation status		
Negative	40 (18.2)	47 (23.6)
*BRCA1*-positive	22 (10.0)	22 (11.1)
*BRCA2*-positive	26 (11.8)	21 (10.6)
Positive (unknown type)	2 (0.9)	0 (0.0)
*BRCA1*- and *BRCA2*- positive	1 (0.5)	0 (0.0)
VUS	2 (0.9)	1 (0.5)
Not tested	127 (57.7)	108 (54.3)
Oophorectomy		
No	217 (98.6)	119 (59.8)
Yes (one or partial removal)	3 (1.4)	11 (5.5)
Yes (both ovaries removed)	0	69 (34.7)
History of high-risk lesion		
No	178 (81.3)	125 (63.1)
Atypical hyperplasia (ADH and ALH)	11 (5.0)	27 (13.6)
LCIS	30 (13.7)	46 (23.2)
Hormonal Medications at the time of MRI		
Oral contraceptives		
No	160 (73.1)	NA
Yes	59 (26.9)	
Menopausal hormone therapy		
No	NA	166 (83.4)
Yes		33 (16.6)
Tamoxifen		
No	214 (97.3)	196 (98.5)
Yes	6 (2.7)	3 (1.5)
Raloxifene		
No	220 (100)	179 (90.0)
Yes	0	20 (10.0)
Aromatase inhibitor		
No	NA	198 (99.5)
Yes		1 (0.5)
Other Exposures at the time of MRI		
Usual alcohol consumption		
None	29 (13.2)	47 (23.6)
<7 drinks per week	170 (77.3)	131 (65.8)
≥7 drinks per week	21 (9.5)	21 (10.6)
Smoking status		
No	207 (94.5)	196 (98.5)
Yes	12 (5.5)	3 (1.5)

### Association between BPE and MRI-FGT with breast cancer risk factors

Table 4 shows results from multivariable-adjusted models for BPE and MRI-FGT in premenopausal women. A positive association between BPE and BMI was observed, but this result did not reach statistical significance (PR = 1.14, 95% CI 0.96, 1.35). There was also a positive association between BPE and use of oral contraceptives at the time of MRI, such that women who reported using oral contraceptives tended to have a higher prevalence of high BPE, however, this finding did not reach statistical significance (PR = 1.45, 95% CI 0.98, 2.15). Conversely, premenopausal women with documented *BRCA* mutations were less likely to have higher BPE than non-carriers (PR = 0.40, 95% CI 0.19, 0.83). It was thought that this relationship could be explained by use of preventive medications (e.g., tamoxifen) in this high-risk group. However, none of the women identified as premenopausal *BRCA* mutation carriers reported use of preventive medications at the time of MRI. In multivariable-adjusted models of MRI-FGT in premenopausal women, BMI was significantly associated with MRI-FGT, with increasing BMI associated with a lower prevalence of high MRI-FGT (PR = 0.66, 95% CI 0.57, 0.77 per five-unit increase in BMI). We also found a significant positive association between having a personal history of LCIS and the prevalence of high MRI-FGT (PR = 1.22, 95% CI 1.02, 1.45).

**Table 4:** Multivariable adjusted prevalence ratios (PR) and 95% confidence intervals (CI) for the relationship between breast cancer risk factors and BPE and MRI-FGT in premenopausal women.

Variable	BPE^a^	*p* value	MRI-FGT^a^	*p* value
Age at MRI^b^ (years)	0.96 (0.82, 1.12)	0.59	0.97 (0.92, 1.03)	0.32
BMI (kg/m^2^)^b^	1.14 (0.96, 1.35)	0.14	0.66 (0.57, 0.76)	<0.0001
Family history of breast cancer				
No	1.00	–	1.00	–
Yes	0.94 (0.59, 1.50)	0.81	1.01 (0.87, 1.17)	0.90
*BRCA* mutation status				
Negative	1.00	–	1.00	–
Positive	0.40 (0.19, 0.83)	0.01	0.94 (0.77, 1.14)	0.53
Not tested	0.94 (0.61, 1.45)	0.78	0.96 (0.82, 1.13)	0.64
History of breast biopsy				
No	1.00	–	1.00	–
Yes	0.98 (0.65, 1.47)	0.91	1.07 (0.94, 1.21)	0.30
History of high-risk benign lesion				
No	1.00	–	1.00	–
Atypical hyperplasia	1.25 (0.59, 2.65)	0.56	0.64 (0.36, 1.16)	0.14
LCIS	0.77 (0.37, 1.62)	0.50	1.22 (1.02, 1.45)	0.03
Age at menarche				
<13 years	1.00	–	1.00	–
≥13 years	0.88 (0.61, 1.27)	0.50	0.97 (0.86, 1.10)	0.68
Number of full-term pregnancies				
Nulliparous	1.00	–	1.00	–
1	0.91 (0.44, 1.85)	0.79	1.04 (0.83, 1.31)	0.70
2	1.25 (0.78, 2.00)	0.36	1.04 (0.89, 1.20)	0.64
≥3	1.32 (0.76, 2.29)	0.33	1.03 (0.86, 1.23)	0.76
Age first full-term pregnancy (years)				
Nulliparous	1.00	–	1.00	–
<25	1.36 (0.64, 2.88)	0.42	1.15 (0.73, 1.81)	0.54
25–<30	1.22 (0.72, 2.07)	0.47	1.11 (0.96, 1.29)	0.17
≥30	1.16 (0.72, 1.88)	0.54	0.99 (0.85, 1.16)	0.91
Use of oral contraceptive at the time of MRI				
No	1.00	–	1.00	–
Yes	1.45 (0.98, 2.15)	0.07	1.01 (0.89, 1.14)	0.92
Use of preventative medications at the time of MRI				
No	–	–	1.00	–
Yes	–	–	0.93 (0.54, 1.60)	0.80
Usual alcohol consumption at the time of MRI				
None	1.00	–	1.00	–
<7 drinks per week	0.98 (0.56, 1.74)	0.95	1.00 (0.83, 1.20)	0.98
≥7 drinks per week	1.48 (0.75, 2.94)	0.26	1.01 (0.80, 1.29)	0.90
Smoking status at the time of MRI				
No	1.00	–	1.00	–
Yes	1.01 (0.40, 2.54)	0.98	1.14 (0.87 1.50)	0.35

^a^PR (95% CI) adjusted age, BMI, family history of breast cancer, *BRCA* mutation status, history of biopsy, history of high-risk benign lesion, age at menarche, number of full-term pregnancies, use of oral contraceptives at time of MRI, number of drinks per week, and smoking status. BPE is categorized as minimal and mild versus moderate and marked. MRI-FGT is coded as fatty and scattered versus heterogeneously dense and dense.

^b^Per 5 unit increase in age (years) and BMI (kg/m^2^), respectively.

In postmenopausal women (Table 5), BPE tended to be lower with increasing age, although this did not reach statistical significance (p = 0.09). A significant positive association between BMI and BPE was observed such that each five-unit increase in BMI was associated with a 44% higher prevalence of high BPE (PR = 1.44, 95% CI 1.08, 1.93). Compared to women who were nulliparous, those who were 30 years or older at the time of first full-term pregnancy had a lower prevalence of high BPE (PR = 0.33, 95% CI 0.13, 0.86). Only BMI was significant associated with MRI-FGT in postmenopausal women, with a 34% decrease in the prevalence of high MRI-FGT seen with every five-unit increase in BMI (PR = 0.66, 95% CI 0.56, 0.78) (Table 5).

**Table 5:** Multivariable adjusted prevalence ratios and 95% confidence intervals (CI) the relationship between breast cancer risk factors and BPE and MRI-FGT in postmenopausal women.

Variable	BPE^a^	*p* value	MRI-FGT^a^	*p* value
Age at MRI^b^ (years)	0.78 (0.59, 1.04)	0.09	0.99 (0.91, 1.07)	0.71
BMI (kg/m^2^)^b^	1.44 (1.08, 1.93)	0.01	0.66 (0.56, 0.78)	<0.0001
Family history of breast cancer				
No	1.00	–	1.00	–
Yes	1.51 (0.47, 4.78)	0.49	1.13 (0.85, 1.49)	0.41
*BRCA* mutation status				
Negative	1.00	–	1.00	–
Positive	0.44 (0.13, 1.48)	0.18	0.86 (0.58, 1.28)	0.45
Unknown	0.58 (0.26, 1.31)	0.19	1.04 (0.79, 1.39)	0.76
History of breast biopsy				
No	1.00	–	1.00	–
Yes	1.49 (0.47, 4.71)	0.50	1.09 (0.82, 1.47)	0.54
History of high-risk benign lesion				
No	1.00	–	1.00	–
Atypical hyperplasia	1.12 (0.34, 3.69)	0.85	0.97 (0.71, 1.33)	0.87
LCIS	1.42 (0.45, 4.46)	0.54	1.12 (0.79, 1.58)	0.52
Age at menarche				
<13 years	1.00	–	1.00	–
≥13 years	0.89 (0.39, 2.02)	0.77	0.97 (0.79, 1.19)	0.79
Number of full-term pregnancies				
Nulliparous	1.00	–	1.00	–
1	0.14 (0.02, 1.19)	0.07	1.15 (0.82, 1.60)	0.41
2	0.63 (0.28, 1.44)	0.27	0.81 (0.63, 1.04)	0.10
≥3	0.43 (0.15, 1.22)	0.11	0.93 (0.67, 1.28)	0.64
Age first full-term pregnancy (years)				
Nulliparous	1.00	–	1.00	–
<25	0.96 (0.35, 2.64)	0.94	0.69 (0.44, 1.08)	0.10
25-<29	0.38 (0.13, 1.11)	0.08	0.74 (0.55, 1.01)	0.06
≥30	0.33 (0.13, 0.86)	0.02	1.11 (0.88, 1.41)	0.37
Use of menopausal hormone therapy at the time of MRI				
No	1.00	–	1.00	–
Yes	2.54 (0.80, 8.01)	0.11	0.92 (0.61, 1.40)	0.69
Use of preventative medications at the time of MRI				
No	–	–	1.00	–
Yes	–	–	0.81 (0.57, 1.16)	0.25
Usual alcohol consumption at the time of MRI				
None	1.00	–	1.00	–
≥1 drink(s) per week	0.58 (0.25, 1.33)	0.20	0.95 (0.73, 1.23)	0.68
Smoking status at the time of MRI				
No	1.00	–	1.00	–
Yes	5.63 (0.53, 60.26)	0.15	1.25 (0.29, 5.31)	0.76

^a^PR (95% CI) adjusted age, BMI, family history of breast cancer, *BRCA* mutation status, history of biopsy, history of high-risk benign lesion, age at menarche, number of full-term pregnancies, use of hormone replacement therapy at time of MRI, ever preventative medication use, number of drinks per week, and smoking status. BPE is categorized as minimal and mild versus moderate and marked. MRI-FGT is coded as fatty and scattered versus heterogeneously dense and dense.

^b^Per 5 unit increase in age (years) and BMI (kg/m^2^), respectively.

### Sensitivity analyses

Sensitivity analyses were conducted restricting to women who self-reported White/Caucasian or reported having an MRI for high-risk screening purposes and the results did not differ (results not shown).

### BPE and MRI-FGT and use of preventive medications

Finally, the use of preventive medications was associated with low BPE in both pre- and post-menopausal women (Table 6). The impact of these medications on BPE is so strong that all women using these medications had low BPE (*p* = 0.07 in premenopausal women and *p* = 0.05 in postmenopausal women). We therefore could not include these variables in the multivariable models for BPE due to small (zero) cell counts. No association between current use of preventive medications and MRI-FGT was observed (*p* > 0.05) (Table 6).

**Table 6:** Distribution of BPE and MRI-FGT by preventive medication use at the time of MRI in pre- and postmenopausal women.

BPE	Minimal/Mild, *N* (%)	Moderate/Marked, *N* (%)	*p* value^a^
Premenopausal^b^			
No	138 (64.5)	76 (35.5)	0.07
Yes	6 (100.0)	0 (0.0)	
Postmenopausal			
No	151 (86.3)	24 (13.7)	0.05
Yes	24 (100.0)	0 (0.0)	
MRI-FGT	Fatty/Scattered, *N* (%)	Heterogeneously Dense/Dense, *N* (%)	
Premenopausal			
No	43 (20.1)	171 (79.9)	0.60
Yes	2 (33.3)	4 (66.7)	
Postmenopausal			
No	86 (38.9)	107 (61.1)	0.51
Yes	11 (45.8)	13 (54.2)	

^a^Cochran Mantel Henzel p-value.

^b^Preventive medications used by premenopausal women included tamoxifen only (see Table 3).

## Discussion

BPE and MRI-FGT are characteristics of normal breast tissue that are routinely assessed by radiologists from standard contrast-enhanced MRI. Prior studies have shown these markers to be independently associated with breast cancer risk, contributing distinct information about a woman’s risk^6,16^. To better understand these relationships and how they could be used to inform recommendations for screening and prevention, it is necessary to understand the factors that impact these imaging markers. The results of the current study show that BPE is highly dependent on hormonal exposures, with a positive association observed between BMI (postmenopausal women only) and a strong inverse association with use of preventive medications (e.g., tamoxifen, aromatase inhibitors).

BPE was found to be positively associated with BMI in both pre-and post-menopausal women. This relationship was most clear (and statistically significant) for postmenopausal women where for every 5-unit increase in BMI the prevalence of high BPE increases by about 40%. While a positive association was also observed in premenopausal women, this association did not reach statistical significance. These results are consistent with those of two prior studies observing a positive association between BMI and BPE. In a small study of 214 women, Hellgren et al.^21^ found that women with obesity (BMI > 30 kg/m^2^) had an almost 5-fold higher odds (95% CI 1.2, 19.4) of having high versus low BPE compared to women with a BMI < 25 kg/m^2^. A second study found an association between BMI and BPE in unadjusted analyses^22^. Ours is the largest multivariable-adjusted study to-date, able to also examine this relationship stratified by menopausal status. Consideration of menopausal status is important given the complex relationship between BMI, menopausal status, and breast cancer risk.

BMI is positively associated with breast cancer risk in postmenopausal women and inversely associated with risk in premenopausal women^27^. This relationship is likely explained in part by the relationship between BMI and hormones (e.g., estradiol)^28^, where in postmenopausal women, adipose tissue becomes the primary source of circulating estrogens^29^. There is a growing body of evidence showing BPE to be associated with both endogenous and exogenous hormonal exposures. BPE has been shown to increase with the use of MHT^23,24^ and to decrease with menopause^7^ and oophorectomy^30^ and in response to treatment with tamoxifen^9,25^, or aromatase inhibitors^8,26^. Recently we also showed that BPE is significantly positively associated with serum estradiol levels in postmenopausal women^20^. Together this suggests a plausible mechanism through which BMI could be impacting BPE in postmenopausal women. Further, there is some indication that the relationship between BPE and breast cancer risk may be modified by BMI, however, this requires further investigation^16^.

In the current study, we found a strong relationship between the use of hormonal medications at the time of MRI and BPE. Specifically, the use of preventive medications (e.g., tamoxifen) was so strongly associated with low BPE in both pre- and postmenopausal women, that all women using these medications had low BPE. We also found that use of oral contraceptives at the time of MRI was associated with a higher prevalence of high BPE, however, this association did not reach statistical significance. Prior work has shown that current or recent use of oral contraceptives is associated with increased breast cancer risk^31,32^. These results support the hypothesis that BPE is a reflection of current/recent hormonal exposures (endogenous and exogenous) experienced in the breast, however further investigation of the influence of oral contraceptive use on BPE is warranted.

Prior work has shown a positive association between BPE and MHT^23,24^. We found that use of MHT at the time of MRI was associated with a non-significant increase in the prevalence of high BPE. This is likely due to the small number of women reporting use of MHT, and the high proportion of those reporting use of local therapy. Specifically, of the 33 women who reported MHT use at the time of MRI, 21 reported use of estrogen-only therapy and 16 of these women reported use of local therapy (e.g., Vagifem, Estring). We did not have the sample size to conduct analyses further stratified by subtype, but since it is thought that BPE may be an indicator of local and systemic hormonal exposures this could have important implications for the use of local estrogen therapy in women undergoing treatment for breast cancer with aromatase inhibitors^33^. Notably we did not see similar associations with MRI-FGT.

MPD has been studied extensively, and it is known to be highly variable across the population. Overall it has been shown to decrease with increasing age, increasing the number of births^34–36^, and increasing BMI^34^. MPD has also been shown to be influenced by hormonal exposures, decreasing with menopause, and with tamoxifen use in postmenopausal women^37^, and increasing with administration of combined estrogen and progestin MHT (but not estrogen alone therapy)^38^. Similarly, we observed an inverse relationship between MRI-FGT and BMI. This was expected given that MRI-FGT is a volumetric measure of the amount of fibroglandular tissue in the breast that has been shown to correlate with MPD^4,5,39^. Other factors (e.g., parity, MHT) previously associated with MPD were not associated with MRI-FGT in this study. We hypothesize that this is because the current analysis is focused on exposure status at the time of MRI. Prior work by our group has shown that BPE is highly dynamic and responsive to the hormonal environment of the breast, whereas changes in MRI-FGT take longer to be observed on MRI^8,9^. This supports the idea that BPE may be an imaging biomarker of current hormonal exposures in the breast and could be used as an indicator of response to hormonal medications^40^. One finding that deserves further consideration is the relationship between LCIS and the prevalence of high MRI-FGT. To our knowledge, this association has not been shown before, however breast density has been shown to play an important role in breast cancer risk among women with LCIS^41^.

One unexpected finding was that *BRCA* mutation carrier status was inversely associated with BPE in premenopausal women such that women carrying a *BRCA* mutation were 60% less likely to have high BPE, compared to non-carriers. It was thought that this association could be related to use of preventive medications (prescribed as chemoprevention in this high-risk population), however, none of the premenopausal *BRCA* mutation carriers reported use of these medications. Further, because this association is only observed in premenopausal women, we know that the lower BPE is also not due to prophylactic oophorectomy.

A small study conducted in Chinese women found *BRCA* mutation carriers to have lower BPE than non-carriers in unadjusted analyses^42^. However, the results of this study (while consistent with our findings) are hard to interpret given that it is not clear if this association is due to other differences between carriers and non-carriers (e.g., use of preventive medications, differences in age or menopausal status). To date no studies have specifically addressed the relationship between BPE and breast cancer risk in *BRCA* mutation carriers. However, the few studies that have examined the association between BPE and breast cancer risk, suggest that BPE is also a risk factor for breast cancer in these women^12^.

A major strength of this study is the collection of data on multiple breast cancer risk factors from both a study questionnaire at the time of MRI, and patients’ medical records. This is particularly important for the assessment of the impact of hormonal medications and lifestyle factors (e.g., smoking and alcohol), where in our study current use (at the time of MRI) was assessed. Inter-reader variability is an important consideration in studies involving the assessment of BPE and MRI-FGT, with many ongoing efforts to develop computer-assisted algorithms for image assessment to reduce potential variability. Here we used a single experienced reader, with repeat BPE and MRI-FGT reads showing very high concordance (i.e., ‘almost perfect agreement’). Although the use of a single reader could potentially limit the generalizability of the study, the high degree of reproducibility of repeat reads by the study radiologist shows strong internal validity. Further, by dichotomizing BPE as low (min/mild) and high (moderate/marked), external validity is increased by reducing the potential for discordance.

Further limitations include the lack of racial diversity in the study population, with 89% of women self-identifying as White, limiting our ability to examine the impact of race on BPE or MRI-FGT. We also lacked information on the timing of MRI with respect to week of the menstrual cycle for pre-menopausal women. The American College of Radiology recommends that MRIs be performed in week 2 of the menstrual cycle when BPE is thought to be at its lowest, thereby maximizing the sensitivity of the MRI for cancer detection^43^. Information on menstrual cycle week was not consistently available for study participants and so is not part of the current analysis. The impact of this is likely negligible as recent papers have found no association between menstrual cycle week and BPE^44,45^. Finally, we did not have information on all breast cancer risk factors. Of particular interest could be the relationship between physical activity and BPE. Physical activity is a potentially modifiable risk factor that has been shown to be associated with both circulating hormone levels and body composition^46,47^.

Prior work has found BPE to be a promising new marker of breast cancer risk providing information beyond that provided by assessment of breast density^6,16^. The development of abbreviated MRI screening protocols likely means that MRI will increasingly be used to screen women that do not meet the high-risk criteria. This could include women with dense breasts^48^ or those at higher than average (elevated) risk^49^. This highlights the need to understand the factors that influence BPE. Here we show that BPE is significantly positively associated with BMI in postmenopausal women. Further, use of preventive medications led to an almost complete reduction in BPE. The hormonally responsive nature of BPE, supported by this and prior studies, suggests that BPE could be an imaging biomarker of hormonal exposures in the breast, potentially used as an indicator of response to hormonal medications, or to further stratify women at high risk of breast cancer undergoing MRI screening.

## Methods

This study was reviewed and approved by the Memorial Sloan Kettering Cancer Center (MSK) Institutional Review Board, and written informed consent was obtained at the time of recruitment from all study participants.

### Study population

Patient recruitment and data collection have been published previously^20^. Briefly, women (*N* = 504) who had no prior history of any cancer (including ductal carcinoma in situ [DCIS], but excluding nonmelanoma skin cancer) as noted in their medical record were approached in the MRI screening clinic at MSK between August 2012 and March 2014. Of the 449 women (88.9%) who volunteered to participate in the study, 30 were ultimately determined to be ineligible. Reasons for exclusion included insufficient proficiency in English (*n* = 2), prior personal history of cancer not previously identified during medical record review (*n* = 14), incomplete study questionnaire (*n* = 2) and diagnosis of breast cancer within the six months following MRI (*n* = 12). Individuals missing information on any covariates were also excluded (3 premenopausal women and 1 postmenopausal woman). This left a study population of 415 women for the current analysis.

### Data collection

All women completed a self-administered questionnaire at the time of their MRI capturing information related to reproductive history, use of hormonal medications, family history of breast cancer and other risk factors. These included: age at menarche and menopause, parity (number of full-term pregnancies, age at first full-term pregnancy, time since last full-term pregnancy), use of hormonal medications at the time of MRI (e.g., MHT, tamoxifen, raloxifene, aromatase inhibitors), weight and height at the time of MRI, family history of breast cancer, and history of oophorectomy. Data from questionnaires was confirmed, when possible, through review of medical records.

### Contrast-enhanced MRI and assessment of BPE and MRI-FGT

Breast MRIs were conducted using standard imaging protocols as described previously^20^. BPE and MRI-FGT were assess by a single reader using the proposed American College of Radiology Breast Imaging Reporting and Data System (BI-RADS) criteria^50,51^. As such, BPE was classified as: minimal, mild, moderate or marked, and MRI-FGT as: a. almost entirely fatty, b. scattered fibroglandular tissue, c. heterogeneous fibroglandular tissue, or d. extreme fibroglandular tissue. The radiologist was blinded to all clinical characteristics of the patients.

BPE and MRI-FGT are usually similar between breasts^11^. To confirm this, readings were conducted in both breasts and were only found to be discordant in one individual. In this instance, the higher value of the two breasts was assigned. Finally, to assess agreement between repeat reads, a set of MRIs (*n* = 19) were randomly selected to be re-read for both BPE and MRI-FGT.

### Statistical Analysis

This was a cross-sectional study examining the relationship between BPE, MRI-FGT and breast cancer risk factors. BI-RADS categories of BPE and MRI-FGT were collapsed to create dichotomous variables categorizing BPE as minimal/mild and moderate/marked, and MRI-FGT as predominantly fatty/scattered and heterogeneous/extreme.

Breast cancer risk factors considered included: age at MRI (continuous), menopausal status (see below), current BMI (continuous), first-degree family history of breast cancer (yes, no), personal history of breast biopsy (yes, no), history of high-risk benign lesions (none, atypical hyperplasia (atypical ductal hyperplasia [ADH] or atypical lobular hyperplasia [ALH]), lobular carcinoma in situ (LCIS), age at menarche (<13 years of age, ≥13 years of age, based on the median age at menarche in the study population), parous (yes/no), number of full-term pregnancies (nulliparous, 1, 2, ≥3), and age at first full-term pregnancy (nulliparous, <25, 25–<29, ≥30 years). *BRCA1/2* mutation status categories were collapsed (negative, positive [*BRCA1*-positive, *BRCA2*-positive, *BRCA1*- and *BRCA2*-positive, positive-unknown type, variant of unknown significance (VUS)], untested [Table 3]) because of insufficient numbers in some subgroups. Use of hormonal medications at the time of MRI was also captured and included use of oral contraceptives (premenopausal women only; yes/no), MHT (postmenopausal women only; yes (any type)/no), and preventive medications (current use of tamoxifen, raloxifene, aromatase inhibitors; yes/no). Other exposures including smoking status (yes/no), alcohol consumption (yes/no), and the number of drinks per week (none, <7, ≥7) were also examined.

If a woman reported that she had not had a menstrual period in the previous 12 months or had a personal history of a bilateral oophorectomy she was considered postmenopausal at the time of MRI. Five women were either missing information on age at the last menstrual period (*n* = 2) or had a period between 6 and 12 months of enrollment (*n* = 3). Eleven women, ranging in age from 44 to 64 years reported having had a simple hysterectomy, making it challenging to determine their menopausal status. For these groups of women, a prior analysis in this study population that included serum measurements of estradiol and estrone found that all had hormone levels were within the postmenopausal range. This indicated that they were indeed postmenopausal at the time of MRI^20^.

Mutually adjusted prevalence ratios (PR) and 95% confidence intervals (CI) were estimated using modified Poisson regression (i.e., Poisson regression using a log link and with robust error variance)^52^. Models did not include an adjustment for MRI-FGT because there was no association between BPE and MRI-FGT in our data (thus it did not meet the requirement of a confounder). Further, for analyses related to BMI, there was concern about over-adjustment given the strong relationship between BMI and MRI-FGT. All analyses were conducted stratified by menopausal status. Analyses were also conducted restricting to women who were having an MRI for high-risk screening purposes (i.e., excluding women with an abnormal mammogram or lump, *N* = 348) and then again in those that self-reported White/Caucasian race/ethnicity (*N* = 369).

Concordance between repeat BPE and MRI-FGT reads was assessed using Cohen’s kappa coefficients. All statistical analyses were conducted using SAS 9.4 (SAS Institute Inc., Cary NC) and all *p* values are 2-sided.

## Data Availability

The data that support the findings of this study are available on request from the corresponding author. The data are not publicly available due to privacy or ethical restrictions.
